# Vagus Nerve Stimulation Exerts the Neuroprotective Effects in Obese-Insulin Resistant Rats, Leading to the Improvement of Cognitive Function

**DOI:** 10.1038/srep26866

**Published:** 2016-05-26

**Authors:** Titikorn Chunchai, Bencharunan Samniang, Jirapas Sripetchwandee, Hiranya Pintana, Wanpitak Pongkan, Sirinart Kumfu, Krekwit Shinlapawittayatorn, Bruce H KenKnight, Nipon Chattipakorn, Siriporn C. Chattipakorn

**Affiliations:** 1 Neurophysiology Unit, Cardiac Electrophysiology Research and Training Center, Faculty of Medicine, Chiang Mai University, Chiang Mai, 50200, Thailand; 2 Cardiac Electrophysiology Research and Training Center, Department of Physiology, Faculty of Medicine, Chiang Mai University, Chiang Mai, 50200, Thailand; 3 Emerging Therapies, Cyberonics Inc, Houston, Texas, USA; 4 Department of Oral Biology and Diagnostic Sciences, Faculty of Dentistry, Chiang Mai University, Chiang Mai, 50200, Thailand

## Abstract

Vagus nerve stimulation (VNS) therapy was shown to improve peripheral insulin sensitivity. However, the effects of chronic VNS therapy on brain insulin sensitivity, dendritic spine density, brain mitochondrial function, apoptosis and cognition in obese-insulin resistant subjects have never been investigated. Male Wistar rats (n = 24) were fed with either a normal diet (n = 8) or a HFD (n = 16) for 12 weeks. At week 13, HFD-fed rats were divided into 2 groups (n = 8/group). Each group was received either sham therapy or VNS therapy for an additional 12 weeks. At the end of treatment, cognitive function, metabolic parameters, brain insulin sensitivity, brain mitochondrial function, brain apoptosis, and dendritic spines were determined in each rat. The HFD-fed with Sham therapy developed brain insulin resistance, brain oxidative stress, brain inflammation, and brain apoptosis, resulting in the cognitive decline. The VNS group showed an improvement in peripheral and brain insulin sensitivity. VNS treatment attenuated brain mitochondrial dysfunction and cell apoptosis. In addition, VNS therapy increased dendritic spine density and improved cognitive function. These findings suggest that VNS attenuates cognitive decline in obese-insulin resistant rats by attenuating brain mitochondrial dysfunction, improving brain insulin sensitivity, decreasing cell apoptosis, and increasing dendritic spine density.

Obesity has reached epidemic proportions in many countries around the world^1^. Several studies have shown that obesity leads to the development of insulin resistance^2^,^3^,^4^. It has been shown that insulin resistance is associated with learning impairment and memory decline^5^,^6^. Previous studies from our group and others have demonstrated that obese rats, induced by high-fat diet (HFD) consumption, not only caused peripheral insulin resistance, but also brain insulin resistance, increased pro-inflammatory cytokines, increased brain oxidative stress, brain mitochondrial dysfunction, hippocampal synaptic dysfunction, decreased dendritic spine density, and instigated cognitive decline^2^,^6^,^7^,^8^,^9^,^10^,^11^.

Vagus nerve stimulation (VNS) is commonly used to treat refractory epilepsy^12^. In addition to epileptic therapy, several studies demonstrated that VNS caused weight loss and reduced body fat in rodents^13^,^14^. Weight loss is a secondary outcome in individuals undergoing VNS for the treatment of refractory epilepsy^12^ or depression^15^. The underlying mechanisms of VNS induced weight loss have been proposed to be due to the stimulation of vagal afferent fibers which provided the satiating effects via a gut-brain feedback mechanism, leading to lower food consumption and subsequent weight loss^16^,^17^. Furthermore, previous studies have shown that vagal nerve activity in the dorsal vagal complex plays important roles for the insulin secretion and glucose homeostasis^18^,^19^. However, the effect of VNS on peripheral and brain insulin sensitivity in an obese-insulin resistant model has not been investigated.

VNS has been shown to suppress inflammation and decrease oxidative stress *in vitro* studies^20^,^21^. Furthermore, VNS in model of depression increased neurogenesis and enhanced neural activity in many brain areas involving cognition^22^,^23^. Moreover, VNS in normal rats has been shown to modulate neuronal plasticity and increased dendritic complexity^24^. Those findings suggest that VNS could have the positive effect on the cognition in dementia condition. However, the effect of VNS on cognitive function in an obese-insulin resistant model has not yet been determined.

In the present study, the effects of chronic VNS on brain insulin sensitivity, synaptic plasticity and brain mitochondrial function, neuronal apoptosis and cognitive function were determined in obese-insulin resistant rats induced by HFD consumption. We tested the hypothesis that chronic VNS on the left vagus nerve in obese-insulin resistant rats induced by HFD improves cognitive function by restoring brain insulin sensitivity, attenuating brain mitochondrial dysfunction and enhancing dendritic spine density as well as attenuating neuronal apoptosis.

## Results

### Long-term HFD consumption caused peripheral insulin resistance but was improved by VNS

After 12 weeks of HFD, the body weight, plasma insulin level and HOMA index of HFD-fed rats increased significantly when compared to ND-fed rats (Table 1). These findings suggested that 12-week HFD caused peripheral insulin resistance as indicated by hyperinsulinemia, increased HOMA index and the disruption in metabolic parameters.

After 12 weeks of VNS treatment, VNS-treated rats had a significant decrease in visceral fat, plasma insulin level, HOMA index, area under the curve of the oral glucose tolerance test (AUCg), plasma total cholesterol level, plasma total triglyceride and LDL/VLDL cholesterol level, when compared to the sham group (Table 2). All of these findings suggest that VNS attenuated peripheral insulin resistance via enhancing peripheral insulin sensitivity, and ameliorating the changes of lipid profiles in obese-insulin resistant rats.

### VNS improved brain insulin signaling following long-term HFD consumption

**Figure 1:**
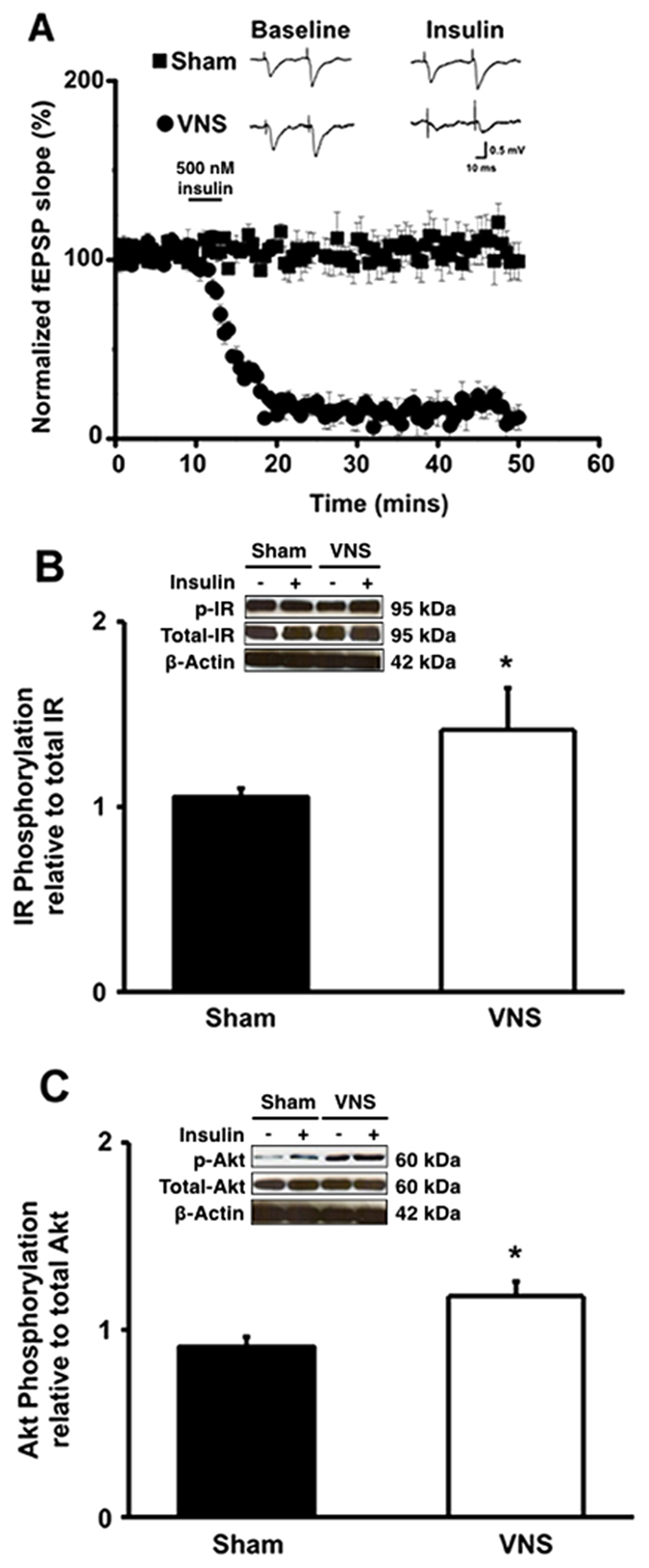
The effects of VNS on brain insulin receptor function, characterized by insulin-induced LTD (**A**), and its signaling, such as the phosphorylation levels of insulin receptor (**B**) and the phosphorylation levels of Akt protein (**C**) Sham: sham-operated HFD-fed rats; VNS: VNS-treated HFD-fed rats (N = 8 of each group) *p < 0.05 in comparison with the sham group.

The insulin-induced long-term depression (insulin-induced LTD) was measured to determine brain insulin receptor function. HFD-fed rats with sham therapy showed impaired insulin-induced LTD, whereas the VNS could reverse this impairment (n = 2–3 independent slices/animal, n = 8 animals/group Fig. 1A). In addition, the expression of insulin receptors (IR) with its phosphorylated form, and the expression of Akt protein with its phosphorylated form were measured to study brain insulin signaling. The phosphorylation of insulin receptors (p-IR) was significantly increased in VNS-treated rats when compared with sham group (Fig. 1B). Similarly, the VNS group had significantly increased phosphorylation of the Akt protein when compared with the sham group (Fig. 1C). All of these findings demonstrated that VNS restored insulin-induced LTD and improved brain insulin signaling that was previously impaired by long-term HFD consumption.

### VNS attenuated brain mitochondrial dysfunction in obese-insulin resistant rats

**Figure 2:**
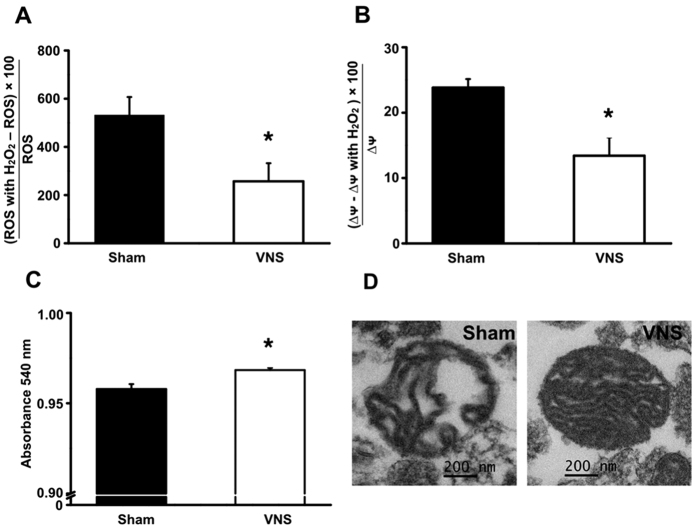
The effects of VNS on brain mitochondrial function in obese-insulin resistant rats, including brain mitochondrial ROS production (**A**), brain mitochondrial membrane potential changes (**B**) and brain mitochondrial swelling (**C**). Representative images demonstrate the morphology of brain mitochondria using transmission electron microscopy at the original magnification of 25,000x (**D**) Sham: sham-operated HFD-fed rats; VNS: VNS-treated HFD-fed rats (N = 8 of each group) *p < 0.05 in comparison with the sham group.

Brain mitochondrial dysfunction was characterized by increased of brain mitochondrial ROS production, brain mitochondrial membrane potential depolarization and brain mitochondrial swelling^7^,^8^,^9^. Our results demonstrated that long-term HFD consumption induced brain mitochondrial dysfunction (Fig. 2A–C). In addition, the unfolding of cristae in the brain mitochondria from HFD-fed rats with sham therapy was observed, indicating brain mitochondrial swelling (Fig. 2D). In VNS-treated rats, there was a significant reduction in brain mitochondrial ROS production, brain mitochondrial membrane potential changes and brain mitochondrial swelling when compared with the sham group (Fig. 2). All of these findings suggest that VNS attenuated brain mitochondrial dysfunction caused by long-term HFD consumption.

### VNS attenuated inflammation, brain oxidative stress and cell apoptosis

**Figure 3:**
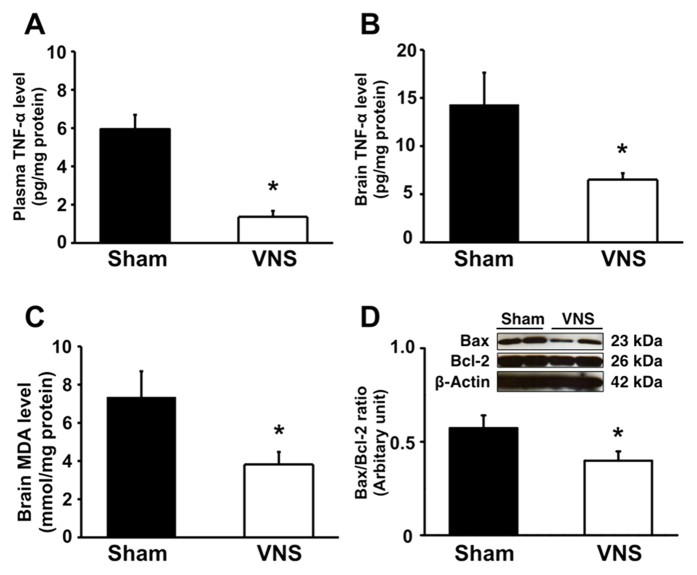
The effects of VNS on inflammation: plasma TNF-α level (**A**) and brain TNF-α level (**B**), brain MDA levels using HPLC system (**C**) and brain apoptosis including Bax/Bcl-2 ratio using western blot analysis (**D**) Sham: sham-operated HFD-fed rats; VNS: VNS-treated HFD-fed rats (N = 8 of each group) *p < 0.05 in comparison with the sham group.

To examine the anti-inflammatory effects of VNS, plasma and brain TNF-α level were determined. After 12 weeks of HFD consumption, the plasma TNF-α level was significantly increased when compared with baseline (5.03 ± 1.21 and 0.27 ± 0.15 pg/mg protein in 12-week HFD vs baseline, p < 0.05). After 12 weeks of VNS treatment, VNS-treated rats had significantly decreased TNF-α levels in both plasma (Fig. 3A) and brain (Fig. 3B) when compared with sham group. All of these findings suggest that VNS attenuated the systemic inflammation, as indicated by decreased plasma and brain TNF-α levels. Moreover, HFD-fed rats with sham therapy demonstrated brain oxidative stress as indicated by an increased brain MDA level (Fig. 3C). VNS treatment could ameliorate brain MDA levels, suggesting an anti-oxidant role of VNS.

The degree of cell apoptosis was also determined in the present study by measuring cell apoptotic markers, including the end product of the apoptotic marker Bax and the anti-apoptotic marker Bcl-2 levels. After 12 weeks of VNS, Bax expression significantly decreased when compared with the sham group. Although there were no significant difference in Bcl-2 levels between the groups, the Bax/Bcl-2 ratio of VNS-treated rats was significantly decreased when compared with sham group (Fig. 3D). All of these findings suggest that VNS also exerted anti-apoptotic effects.

### VNS treatment improved dendritic spine density and cognitive function in obese-insulin resistant rats

**Figure 4:**
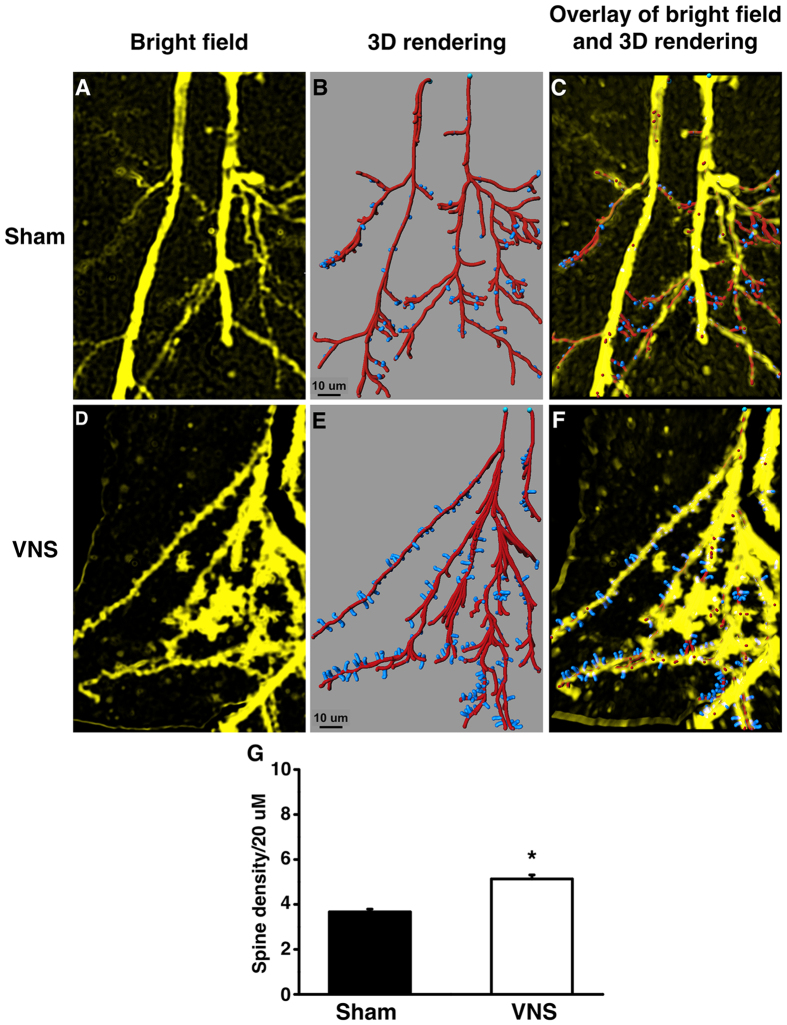
The effects of VNS on dendritic spine density. The representative images from invert bright field of dendritic spines (**A**,**D**) The representative 3D rendering of dendritic spine density was constructed using the Filament Tracer module of the Imaris Suite 7.0^®^ software to generate spine reconstructions of the acquired invert bright field z-stack images (**B**,**E**) The representative overlay images from invert bright field of dendritic spines and 3D rendering (**C**,**F**) Upper panel (**A–C**) showed the representative dendritic spine density of sham group. Lower panel (**D–F**) showed the representative dendritic spine density of VNS group. (**G**) The effect of VNS or sham on the number of dendritic spines at CA1 region of hippocampus. Sham: sham-operated HFD-fed rats; VNS: VNS-treated HFD-fed rats (N = 8 of each group) *p < 0.05 in comparison with the sham group.

There was a loss of dendritic spine density after HFD consumption in sham therapy (Fig. 4A–C). However, chronic stimulation of the vagus nerve increased dendritic spine density (Fig. 4D–F). The number of dendritic spines on the tertiary segment of the apical dendrites in VNS-treated rats was significantly increased when compared with the sham group (Fig. 4G), suggesting that VNS enhanced synaptic strength.

**Figure 5:**
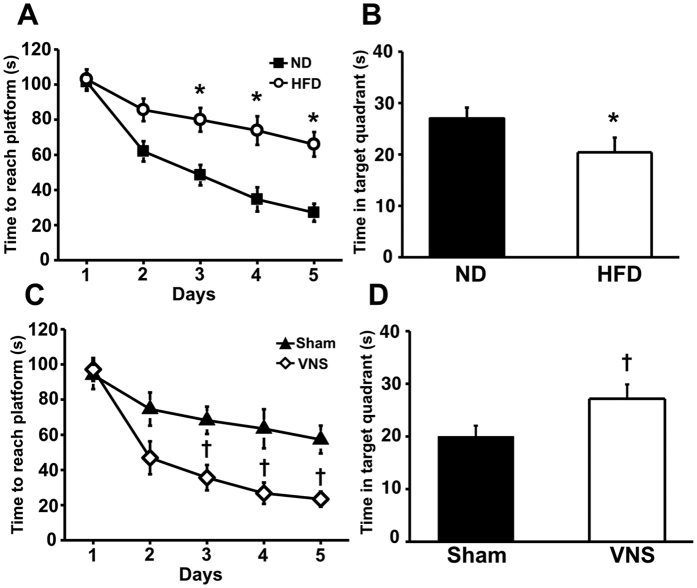
Learning and memory behavioral results from Morris Water Maze test. Chronic HFD consumption caused cognitive impairment as indicated by increased time required to reach the platform in the acquisition test (**A**) and decreased time spent in the target quadrant in the probe test (**B**). VNS treatment attenuated cognitive decline, as shown by decreased time required to reach the platform (**C**) and increased time spent in the target quadrant (**D**). ND: 12-week-normal diet-fed rats; HFD: 12-week high fat-fed rats; Sham: sham-operated HFD-fed rats; VNS: VNS-treated HFD-fed rats (N = 8 of each group) *p < 0.05 in comparison with the ND-fed rats; ^†^p < 0.05 in comparison with the sham group.

As previously demonstrated, long-term HFD consumption could lead to the induction of cognitive impairment in association with a reduction in dendritic spine density^10^. Consistent with a previous study, HFD consumption in the present study also caused memory impairment indicated by the increased time to reach the platform (Fig. 5A) and decreased time spent in the target quadrant in 12-week HFD-fed rats, compared to 12-week ND-fed rats (Fig. 5B). After VNS for 12 weeks in HFD-fed rats, the time to reach the platform in VNS-treated rats was significantly decreased when compared to sham group during the acquisition test (Fig. 5C). In addition, the time spent in the target quadrant during the probe test in VNS-treated rats was significantly higher than that of the sham group (Fig. 5D). All of these findings suggested that VNS effectively attenuates the impairment of learning and memory behaviors caused by long-term HFD consumption.

## Discussion

The major findings of the present study are as follows: 1) The obese condition following long-term HFD consumption led to the development of peripheral insulin resistance, brain insulin resistance, brain mitochondrial dysfunction and cognitive decline possibly through the induction of brain oxidative stress, brain inflammation, brain apoptosis and the reduction of dendritic spine density, and 2) VNS attenuated the undesirable effects of long-term HFD, leading to the improvement of cognitive function.

In the present study, the development of peripheral insulin resistance following long-term HFD consumption was clearly shown as indicated by an increase in the HOMA index, impaired oral glucose tolerance test (OGTT), and metabolic disturbances^7^,^9^,^25^,^26^. It is known that both oxidative stress and inflammation play an important role in the development of insulin resistance^27^. Consistent with this notion, the obese-insulin resistant rats in this study also had an increase in the pro-inflammatory cytokine TNF-α, and oxidative stress.

VNS is known to exert anti-inflammatory effect, and has been shown previously to decrease TNF-α in traumatic brain injury^21^,^28^. Our results demonstrated that VNS therapy provided the anti-inflammatory effect as evidenced by the decreased TNF-α level in obese-insulin resistant rats. Moreover, the present study also demonstrated that VNS therapy effectively decreased oxidative stress. These beneficial effects of VNS could contribute to the increased peripheral insulin sensitivity under this obese-insulin resistant condition. A clinical report in patients with impaired glucose tolerance demonstrated that VNS significantly reduce two-hour glucose tolerance in these patients^29^, thus supporting our findings regarding the benefits of VNS on improved insulin sensitivity.

The present study also demonstrated that long-term HFD consumption not only caused peripheral insulin resistance, but also led to the development of brain insulin resistance, as indicated by the decreased insulin-induced LTD, the reduction of brain insulin signaling, the impaired brain mitochondrial function, and an increased apoptosis in the brain. These findings were consistent with our recent reports in which brain insulin resistance and brain mitochondrial dysfunction was developed after long-term HFD consumption^7^,^9^,^25^. It has been shown that obesity induced cell apoptosis by increased Bax level, decreased Bcl-2 level and impaired brain mitochondrial function^30^. Although previous studies demonstrated that VNS exerted protective effects on mitochondrial function in the heart^31^,^32^, its effect on brain mitochondrial function has never been tested. In the present study, we demonstrated for the first time that VNS effectively attenuated brain insulin resistance, brain mitochondrial dysfunction, and brain apoptosis. Moreover, we found that VNS therapy also decreased brain MDA levels in these obese-insulin resistant rats, indicating that VNS could effectively decrease oxidative stress not only in the plasma, but also in the brain. Our findings suggested that a reduction of brain mitochondrial dysfunction and brain oxidative stress by VNS therapy could be mainly responsible for the neuroprotective effects of VNS in these obese-insulin resistant rats.

Growing evidence demonstrated the potential roles of VNS on cognition in the models of epilepsy and depression^33^,^34^. A recent study by Peña and colleagues demonstrated that VNS facilitated cognition through promoting plasticity and preserving electrical-induced LTD in normal rats^35^. In the present study, we found that VNS could improve cognitive function and increase the dendritic spine density which were all impaired in obese-insulin resistant rats. The effects of VNS to decrease peripheral insulin resistance, brain inflammation, brain mitochondrial dysfunction, brain oxidative stress, brain apoptosis, and to increase brain insulin sensitivity could be responsible for the prevention of the dendritic spine density reduction, and thus leading to an improved cognition as observed in the obese-insulin resistant rats in the present study.

## Conclusion

Obese-insulin resistant condition causes brain insulin resistance, brain oxidative stress and inflammation, and brain apoptosis, resulting in the cognitive decline. VNS exerts the neuroprotective effects in obese-insulin resistant rats via its anti-inflammation, anti-oxidation, anti-apoptosis, as well as its ability to act as an insulin sensitizer, leading to the improvement of cognitive function. This study also demonstrated that VNS is not only effective and safe therapeutic method, but also provides beneficial effects on cognition.

## Methods

### Animal models and experimental protocols

**Figure 6:**
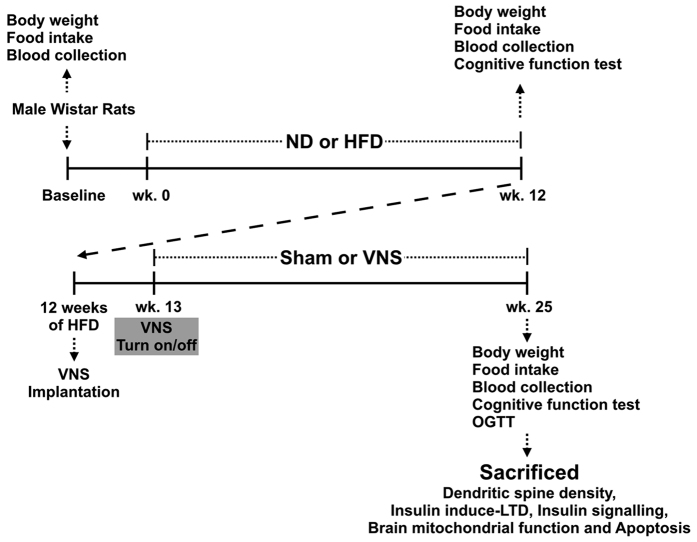
The experimental protocol of the present study.

The methods were carried out in accordance with the approved guidelines. All experimental protocols were approved by the Institutional Animal Care and Use Committee (IACUC) of the Faculty of Medicine, Chiang Mai University (Permit number: 34/2557) and conformed to the Guide for the Care and Use of Laboratory Animals published by the US National Institutes of Health (NIH guide, 8^th^ edition, 2011). Male Wistar rats (n = 24) were used in the present study, and obtained from the National Laboratory Animal Center, Salaya Campus, Mahidol University, Thailand. All rats were housed individually in a temperature-controlled environment (25 ± 0.5 °C) with a 12:12 hour light-dark cycle. After acclimatization for 1 week, rats were divided into two groups, those on a normal diet (ND; n = 8) and those on a high-fat diet (HFD; n = 16). In the ND group, rats were fed on a standard laboratory pelleted diet containing 19.77% fat, 51.99% carbohydrate and 28.24% protein (Mouse Feed Food No. 082, C.P. Company, Bangkok, Thailand) and the rats in the HFD group were fed on a diet consisting of 59.28% fat, 14.27% carbohydrate and 26.45% protein to create the obese-insulin resistant models^2^. After 12 weeks all rats were evaluated for cognitive function using the Morris Water Maze test. The HFD-fed rats were then randomly assigned to either the sham-operated group (Sham; n = 8) or the vagus nerve stimulation (VNS; n = 8) group. After a week of recovery, rats were continuously fed with a HFD for an additional 12 weeks. At week 25, the cognitive function and the oral glucose tolerance test (OGTT) of each rat were investigated. Blood samples were collected from a tail vein at week 12, and week 25 of the experimental protocol for plasma analysis. At the end of the experimental protocol, rats were deeply anesthetized with isoflurane and killed by decapitation. The brain of each rat was quickly removed and carefully sliced in preparation for investigation, including extracellular recording (insulin-induced LTD), immunoblot analysis, brain mitochondrial function and brain MDA measurement. The experimental protocol is summarized in Fig. 6.

### Surgical procedure

Animals were randomized to receive either inactive VNS implantation, or active VNS implantation. All rats were anesthetized with xylazine (3 mg/kg, g, Laboratorios Calier, Barcelona, Spain) and zolitil (50 mg/kg, Virbac Laboratories, Carros, France)^36^. The hair was shaved and skin was cleaned on each rat before the bipolar cuff electrode was implanted around the left cervical vagus nerve and connected to a VNS Demipulse^™^ (Cyberonic, Inc., Houston, Texas). Animals were closely monitored and subcutaneously injected with both 10 mg/kg of marboflxacine and 16 mg/kg of tolfenamic acid^36^. Surgical wounds were bathed with povidine solution as necessary. The VNS group experienced a continuous 14 s delivery of stimulation at a frequency of 20 Hz, pulse width of 500 μs and a current of 0.5–0.75 mA followed by a 48 s rest. The same procedures, without stimulation, were performed in the sham group. For the 10 minutes preceding data collection, VNS therapy was turned off and remained off throughout the duration of the acquisition test and probe trial test.

### Blood sample assays

Plasma glucose and cholesterol levels were determined via colorimetric assay (Biotech, Bangkok, Thailand). The commercial colorimetric assay kit (Biovision, California, USA) was used for determining plasma HDL and LDL levels. Plasma insulin levels were also determined using the Sandwich ELISA kit (LINCO Research, MO, USA). Homeostasis Model Assessment (HOMA) was used for assesse the peripheral insulin resistance as described in previous studies^37^,^38^.

### Oral glucose tolerance test (OGTT)

OGTT was performed as described in Pintana *et al.*^7^,^9^. Briefly, rats were fasted overnight before the test and received 2 g/kg of glucose solution via oral gavage feeding. Blood samples were collected from the tail vein at 0, 15, 30, 60, 90 and 120 minutes after glucose administration. Areas under the curve (AUC) were calculated to evaluate glucose tolerance.

### Brain slice preparation

At the end of experimental period, the rats were anesthetized with isoflurane and decapitated. As described previously^2^,^8^,^9^, the brain tissue in each rat was removed and immersed in ice-cold artificial cerebrospinal fluid (aCSF) containing high sucrose for 30 minutes. Brain slices (400 μm) were cut on a vibratome (Vibratome Company, MO, USA). The slices were transferred to a room temperature (22–24 °C) CSF solution for an additional 30 minutes.

### Extracellular recordings of hippocampal slices for insulin-induced long-term depression (LTD)

Insulin-induce long-term depression (LTD) was examined using the method described in Pratchayasakul et al.2 and Pipatpiboon *et al.*^2^,^8^,^9^. Briefly, the hippocampal slices were transferred to a recording chamber containing standard aCSF. Field excitatory postsynaptic potentials (fEPSPs) were evoked using a bipolar tungsten electrode to stimulate the Schaffer collateral-commissural pathway, while the fEPSPs recordings were taken by micropipettes (3 MΩ) filled with 2 M NaCl from the stratum radiatum in CA1 region of hippocampus. Frequency of the stimulation was 0.033 Hz and the stimulus intensity was adjusted to produce <50% of maximal monophasic responses with 0.8–1.0 mV in amplitude. The brain slices were perfused with aCSF (as the baseline condition) for 10 minutes, followed by perfusion with 500 nM insulin in aCSF (as insulin-induced LTD) for an additional 10 minutes. Thereafter, the slices were perfused with aCSF again for a further 50 minutes. Data were digitized and recorded in a computer using pClamp9.2 software (Axon Instruments, CA, USA). The initial of the fEPSP slope was measured and plotted against time.

### Golgi staining and Image analysis

To examine dendritic spine density, Golgi staining was performed following Sripetchwandee *et al.*^10^. Briefly, the brain was transferred to Golgi staining solution according to FD Rapid Golgistain™ Kits (FD Neuro Technologies, Baltimore, MD, USA) and cut at 60 μm of thickness using Cyostat (Leica CM1950, Leica Biosystem Nussloch GmbH, Nussloch, Germany). For analysis, the three segments, 100–200 μm apart from the soma and 20–30 μm in dendritic length were used to randomly measure dendritic spine density. Three neuronal cells per brain slice, and three brain slices per animal were chosen for quantitative analysis. The number of spines was counted by double-blind hand counter, and the dendritic length was measured using Xcellence imaging software (Olympus). The representative of dendritic spine density was constructed using the Filament Tracer module of the Imaris Suite 7.0^®^ software (Bitplane, Oxford instruments, Concord, MA, USA).

### Immunoblotting for brain insulin signaling and apoptosis

To investigate the brain insulin signaling, homogenate brain slices were used, as described in references^2^,^8^,^9^. IR tyrosine phosphorylation was electrophoresed and an immunoblot analysis was carried out with rabbit antibodies. Examination of the level of IR protein expression was conducted with homogenates prepared from another set of four whole brain slices. These proteins were separated and identified by an immunoblot assay conducted with rabbit anti-IR at tyrosine phosphorylation (1:1000; sc-25103-R; Santa Cruz Biotechnology, CA, USA), IR (1:1000; sc-711; Santa Cruz Biotechnology, CA, USA). Similarly, the Akt phosphorylation were electrophoresed and immunoblotted with rabbit antibodies for Akt (1:1000; 9272S; Cell Signaling Technology, MA, USA). For a loading control, immunoblotting for each membrane was incubated with anti-β-actin (1:4000; #4967; Cell Signaling Technology, MA, USA). All membranes for visualizing the phosphorylation and the protein levels of IR expression were incubated with a secondary goat anti-rabbit antibody conjugated with horseradish peroxidase (1:2000; #7074; Cell Signaling Technology, MA, USA). The protein bands were visualized on Amersham hyperfilm ECL (GE Healthcare, Buckinghamshire, UK) using Amersham ECL Western blot detection reagents (GE, Healthcare). The band intensity was measured by Scion Image, and the results were represented as average signal intensity (arbitrary) units.

### Brain malondialdehyde (MDA) level

To examine the brain oxidative stress, brain malondialdehyde (MDA) level was determined by high performance liquid chromatography (HPLC), as described in the previous studies^7^,^25^.

### Brain mitochondrial function

Brain mitochondria were isolated as described in Pipatpiboon *et al.*^8^. Mitochondrial protein was determined by the BCA assay as described previously^7^, and brain mitochondrial function including brain mitochondrial reactive oxygen species (ROS), mitochondrial membrane potential change (ΔΨm) and mitochondrial swelling were determined^7^,^8^,^9^. Brain mitochondrial reactive oxygen species (ROS) were measured using dichloro-hydrofluoresceindiacetate (DCFHDA) fluorescent dye. The change in mitochondrial membrane potential (ΔΨm) was measured using the fluorescent dye 5, 5′, 6, 6′-tetrachloro-1, 1′, 3, 3′-tetraethyl benzimidazolcarbocyanine iodide (JC-1) and brain mitochondrial swelling was determined by measuring the change in the absorbance of brain mitochondrial suspension at 540 nm. All were determined by following the methods described previously^8^,^25^.

### Cognitive function test

The Morris Water Maze (MWM) test was performed determining cognitive function with two assessments, including five consecutive days of the acquisition test, and the probe test on day sixth. Time to find the platform was recorded in the acquisition test and the time spent in the target quadrant was also recorded in the probe test^25^,^39^. Data analysis of the MWM test was done manually from video tape recordings by the investigators, who were blinded to experimental groups.

### Statistical analysis

Data from each experiment were expressed as mean ± S.E.M. For all comparisons, the significance of the difference in peripheral biochemical parameters, the percentage of insulin-induced LTD, brain mitochondrial function, immunoblot and probe test in the MWM tests were calculated using an independent t-test. Comparisons between the groups of the MWM tests for the acquisition test were performed using a two-way ANOVA, followed by post-hoc LSD analysis. A *P* < 0.05 was considered as statistically significant.

## Additional Information

**How to cite this article**: Chunchai, T. *et al.* Vagus Nerve Stimulation Exerts the Neuroprotective Effects in Obese-Insulin Resistant Rats, Leading to the Improvement of Cognitive Function. *Sci. Rep.* **6**, 26866; doi: 10.1038/srep26866 (2016).

## Figures and Tables

**Table 1:** The metabolic parameters after 12-week of ND or HFD consumption.

Metabolic parameters	12-week ND	12-week HFD
Body weight (g)	475.55 ± 7.92	544.34 ± 5.83*
Food intake (g/day)	21.90 ± 1.30	21.15 ± 0.87
Plasma glucose (mg/dl)	131.25 ± 6.82	127.47 ± 5.04
Plasma insulin (ng/ml)	2.76 ± 0.34	5.87 ± 0.55*
HOMA index	23.76 ± 5.74	33.83 ± 4.53*

^*^p < 0.05 in comparison with ND group.

**Table 2:** The metabolic parameters after 12-week of VNS treatment in comparison with the sham group.

Metabolic parameters	12 weeks post-treatment	
	Sham	VNS
Body weight (g)	613.34 ± 8.97	594.17 ± 10.83
Food intake (g/day)	19.28 ± 1.61	19.46 ± 1.22
Visceral fat (g)	61.01 ± 2.61	51.92 ± 3.53*
Plasma glucose (mg/dl)	131.47 ± 9.60	134.46 ± 3.98
Plasma insulin (ng/ml)	4.85 ± 1.36	3.04 ± 0.42*
HOMA index	30.93 ± 10.85	18.12 ± 2.37*
Plasma glucose AUC (AUCg) (mg/dl × min × 10^4^)	5.58 ± 0.27	4.70 ± 0.23*
Plasma total cholesterol (mg/dl)	105.93 ± 6.34	81.42 ± 2.66*
Plasma total triglyceride (mg/dl)	28.73 ± 3.05	19.64 ± 1.60*
HDL cholesterol (mg/dl)	27.94 ± 1.67	27.39 ± 0.95
LDL/VLDL cholesterol (mg/dl)	70.17 ± 5.79	48.62 ± 1.63*

^*^p < 0.05 in comparison with the sham group.
